# Innate and Adaptive Immunity in Calcific Aortic Valve Disease

**DOI:** 10.1155/2015/851945

**Published:** 2015-05-03

**Authors:** Patrick Mathieu, Rihab Bouchareb, Marie-Chloé Boulanger

**Affiliations:** ^1^Laboratoire d'Études Moléculaires des Valvulopathies (LEMV), Groupe de Recherche en Valvulopathies (GRV), Quebec Heart and Lung Institute/Research Center, Department of Surgery, Laval University, QC, Canada; ^2^Institut de Cardiologie et de Pneumologie de Québec, 2725 Chemin Sainte-Foy, Québec, QC, Canada G1V 4G5

## Abstract

Calcific aortic valve disease (CAVD) is the most common heart valve disorder. CAVD is a chronic process characterized by a pathologic mineralization of valve leaflets. Ectopic mineralization of the aortic valve involves complex relationships with immunity. Studies have highlighted that both innate and adaptive immunity play a role in the development of CAVD. In this regard, accumulating evidence indicates that fibrocalcific remodelling of the aortic valve is associated with activation of the NF-*κ*B pathway. The expression of TNF-*α* and IL-6 is increased in human mineralized aortic valves and promotes an osteogenic program as well as the mineralization of valve interstitial cells (VICs), the main cellular component of the aortic valve. Different factors, including oxidized lipid species, activate the innate immune response through the Toll-like receptors. Moreover, VICs express 5-lipoxygenase and therefore produce leukotrienes, which may amplify the inflammatory response in the aortic valve. More recently, studies have emphasized that an adaptive immune response is triggered during CAVD. Herein, we are reviewing the link between the immune response and the development of CAVD and we have tried, whenever possible, to keep a translational approach.

## 1. Introduction

Ectopic cardiovascular mineralization occurs in different disorders and is associated with a substantial morbidity. Long seen as a passive process ectopic pathologic mineralization is now considered as a highly regulated process at the molecular and cellular levels [1]. Calcific aortic valve disease (CAVD) is a chronic disorder that involves a mineralization of the valvular tissue. Fibrosis often accompanies pathologic mineralization and both processes contribute to the fibrocalcific remodelling of the aortic valve. CAVD is the most common heart valve disease and encompasses a wide variety of clinical presentations. As such, there is a continuum from aortic valve sclerosis to severe aortic stenosis with a common underlying process: ectopic mineralization of the aortic valve [2]. One key central question, still unresolved, is what drives the fibrocalcific remodelling of the aortic valve. Is the mineralization of the aortic valve triggered and enhanced by factors that normally promote the mineralization of bone? Studies performed in the last decade have unravelled important key underlying mechanisms involved in the ectopic mineralization of the aortic valve. These works have highlighted that though there are, to some extent, similarities with the ossification process, the mineralization of the aortic valve relies on specific mechanisms that differ from bone ossification. In fact, the response to different stimuli may diverge considerably in VICs when compared to osteoblasts. These findings may thus explain the association between osteoporosis and ectopic vascular/valvular mineralization [3]. The tissue architecture and specific mechanical strain imposed on the aortic valve drive specific molecular events that may promote pathologic mineralization [4]. Apart from mineralization, key features of CAVD include the abnormal accumulation of different lipid species and inflammatory cells in explanted human pathologic samples [5].

**Figure 1:**
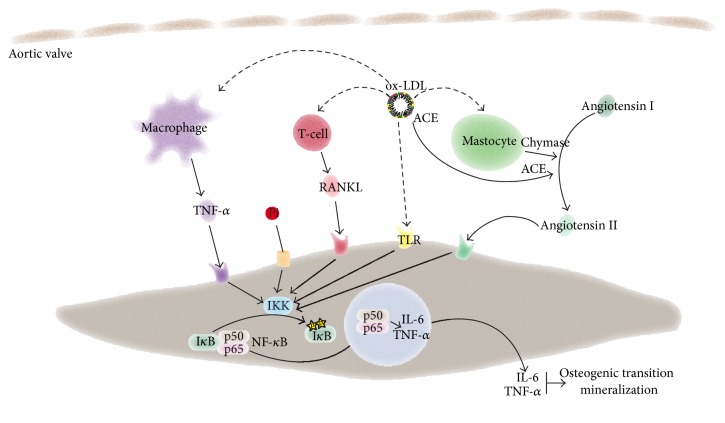
Scheme showing the interaction between inflammatory cells and valve interstitial cells (VICs) and its role during CAVD.

Immunity, both innate and adaptive, has been shown to play an important role in different chronic disorders including atherosclerosis. In CAVD, accumulating evidence clearly indicates that inflammation is involved in the development and possibly in the progression of the disease process [6]. Innate response to different factors has been shown to promote the mineralization of valve interstitial cells (VICs) (Figure 1), the main cellular component of the aortic valve. On the other hand, emerging evidence suggests that specific adaptive immunity could play a substantial role in orchestrating an immune response during CAVD (Figure 1). In this review, we describe, at the molecular and cellular levels, the role of immunity in CAVD.

## 2. Pathobiology of CAVD: An Overview

The aortic valve is normally a thin structure that accomplishes the important task of promoting the unidirectional blood flow from the left ventricle to the aorta. The histological structure of the aortic valve is classically divided into three different layers: the fibrosa, the spongiosa, and the ventricularis. The fibrosa and the ventricularis are the outermost external layers that, respectively, face the aorta and the left ventricle in diastole. The fibrosa mostly contains collagen fibers with dispersed VICs, whereas the ventricularis has a high content of elastic fibers and is populated by both VICs and smooth muscle cells (SMCs). The central layer, the spongiosa, has a high content in glycosaminoglycans (GAGs) and is thought to play an important role in the biomechanical properties of the aortic valve in absorbing a part of the mechanical load during the cardiac cycle [7, 8]. The aortic valve is covered with an endothelium and studies have highlighted that the aortic and ventricular endothelium have different biological properties, which may impact on the development of CAVD [9].

The microscopic analyses of human mineralized aortic valves, obtained from surgeries, have revealed some important key features of CAVD. First, lipids infiltrate the valve tissue often in the vicinity of mineralized areas [10]. Second, dense inflammatory infiltrates are present in some valves where oxidized lipids and ectopic mineralization are observed [6, 11]. Three, in about 15% of mineralized aortic valves osteogenic metaplasia is observed [12]. Coté et al. showed in 285 mineralized valves that the presence of dense chronic inflammatory infiltrates, present in 28% of valves, was associated with osseous metaplasia, neovascularization, and a higher level of tissue remodeling [6]. Furthermore, the density of leukocytes in mineralized aortic valves was associated with a faster progression rate of aortic stenosis. Hence, these data suggest that CAVD has possibly important relationships with inflammation.

## 3. NF-Kappa B Pathway and CAVD

### 3.1. Regulation of the NF-*κ*B Pathway

The nuclear factor-*κ* B (NF-*κ*B) is at the center stage of inflammation. The NF-*κ*B pathway is classically divided into the canonical and noncanonical cascades. The canonical pathway, which is activated by tumor necrosis factor alpha (TNF-*α*) and IL-1*β* among others, relies on activation of the heterodimer formed by p65 (RelA) and p50, which control the expression of several genes involved in the inflammatory response [13]. Upstream, the inhibitor of I*κ*B kinase (IKK), controls the activation of the cascade. IKK is a multisubunit complex, which consists of IKK*α*, IKK*β*, and IKK*γ* (NEMO) [14]. IKK*β* catalytic activity is promoted by IKK*γ*, a regulatory protein that promotes activity of the IKK complex. In turn, the IKK complex phosphorylates I*κ*B*α* on Ser residues 32 and 36. As a result, I*κ*B*α* is targeted for degradation and the p65/p50 heterodimer is liberated from the cytosol and migrates to the nucleus where it controls the expression of target genes [15]. It is worth underlining that posttranscriptional modification of p65 subunit is also important to control the nuclear translocation and transcriptional activities. Twelve phosphosites for p65 have been described so far and have been shown to either enhance or decrease transcriptional activity in a gene-specific and possibly cell-specific manner [16]. For instance, in U937 macrophages stimulated with LPS, Ser 536 phosphorylated p65 is specifically recruited on IL-8 promoter and not on IL-6 and TNF-*α* promoters [17]. Phosphorylation of Ser 276 on p65 by the catalytic unit of protein kinas A (PKAc) has been shown to promote the acetylation of p65 and to allow a stable association with its cofactors CREB-binding protein/p300 and chromatin targets [18]. Reactive oxygen species (ROS) have been shown to act as cofactor in PKAc-mediated phosphorylation on Ser 276 of p65 [19]. Of note, ROS are generated in mineralized aortic valves and promote the expression of osteogenic genes [20]. On the other hand, the noncanonical pathway relies on the phosphorylation of IKK*α* by its upstream kinase NF-*κ*B-inducing kinase (NIK/MEKK14) and is independent from IKK*γ* [21]. Activation of this pathway by different signals, such as lymphotoxin B, CD40L, or B cell activating factor, promotes the nuclear translocation of RelB/p52 [22].

### 3.2. Phosphate and Activation of the NF-*κ*B Cascade

By producing a high level of cytokines VICs play a crucial role in the regulation of inflammation. In explanted human mineralized aortic valves the levels of phosphorylated I*κ*B (Ser 32) are increased significantly [23]. One fact, which is often underappreciated, is that phosphate (Pi) is an important promoter of inflammation in isolated VICs [23]. Phosphates are produced in fairly good amounts by VICs during mineralization owing to a high expression of nucleotide metabolizing enzymes, the ectonucleotidases [24]. During the mineralization of the aortic valve, alkaline phosphatase (ALP) and ectonucleotide phosphodiesterase/pyrophosphatase-1 (ENPP1) are overexpressed. The ectonucleotidases generate Pi at the cell membrane [25–27]. In turn, Pi is internalized by VICs through the sodium-dependent phosphate cotransporter Pit1 (SLC20A1), which is overexpressed in stenotic aortic valves [28]. Though the molecular details are lacking, the intracellular channelling of Pi is associated with a lower level of Akt, a kinase involved in cell survival. We recently identified that Akt prevents the activation of the NF-*κ*B pathway in VICs [23]. Also, the overexpression of ectonucleotidases contributes to the depletion of the extracellular levels of nucleotides, which, in turn, decrease the signalling through the P2Y2 receptor (P2Y2R) [24]. In VICs, secretion of ATP gives a survival signal through the P2Y2R and Akt. Hence, a lower signalling through Akt promotes a sustained activation of the NF-*κ*B pathway. The mechanism whereby Akt decreases NF-*κ*B signalling in VICs remains to be fully investigated, but overexpression of Akt in VICs prevents the nuclear translocation of NF-*κ*B-p65 in response to phosphate and inhibits the mineralization of cell cultures [23].

### 3.3. IL-6 Regulates the Mineralization of the Aortic Valve

**Figure 2:**
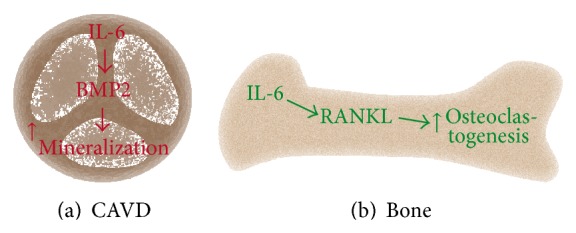
The calcification paradox could be explained by interleukin-6 (IL-6), which mediates the mineralization of the aortic valve and in bone activates osteoclastogenesis.

One important target of NF-*κ*B-p65 is interleukin-6 (IL-6), a pleiotropic cytokine often used as a blood plasma marker of inflammation and cardiovascular risk [29]. However, little is known on the role of IL-6 in CAVD. By using a whole-genome transcriptomic approach El Husseini et al. identified that, among the family of cytokines that signal through gp130, IL-6 is highly expressed in mineralized aortic valves [23]. Evidence indicates that the NF-*κ*B pathway is activated in human explanted aortic valves and thus directly contributes to the secretion of IL-6. IL-6 is produced and secreted in large amounts by VICs. As highlighted above several factors may contribute to promote the production of IL-6 by VICs. In this regard, a high activity of ectonucleotidases contributes to decreasing the signalling through Akt, which in VICs normally represses the NF-*κ*B pathway [23]. Moreover, although not studied in VICs, it is possible that other factors including cytokines such as TNF-*α* and oxidized lipid species may also contribute to trigger the secretion of IL-6. In turn, IL-6 exerts an important control over the osteogenic transition of VICs through auto/paracrine effects. In this regard, silencing of IL-6 in VICs prevented phosphate-induced mineralization of cell cultures [23]. Moreover, the silencing of IL-6 in VICs abrogated the rise of osteogenic transcripts following a treatment with the phosphate-containing medium [23]. Conversely, treatment of VICs with exogenous IL-6 increased the expression of osteogenic genes severalfold. BMP2 is the gene which is the most highly upregulated following a treatment of VIC cultures with IL-6. Inhibition of BMP2 with Noggin, an antagonist of BMP2 and BMP4, prevented IL-6-induced mineralization of VICs [23]. Taken together, a high production of IL-6 by VICs is conducive to an osteogenic transition and mineralization of the aortic valve through a BMP2 pathway [23]. Recently, it has been identified that IL-6 promotes in the aortic valve the endothelial-mesenchymal transition (EndoMT) [30]. Hence, in the aortic valve it is possible that IL-6 increases the amount of mesenchymal cells with osteogenic properties through the EndoMT process. Of interest, in bone, IL-6 stimulates osteoclast activity. Of note, this effect is indirect as it needs the presence of osteoblasts. In this regard, IL-6 induces the expression of receptor activator of nuclear factor-kappa B ligand (RANKL) by osteoblasts whereby osteoclastogenesis is promoted [31]. Chronic and systemic elevation of IL-6 is one important feature of several clinical conditions, including obesity, which are linked with osteoporosis and a higher incidence of CAVD [32]. Hence, though it remains to be investigated it is possible that IL-6 explains, at least in part, the so-called calcification paradox, where bone demineralization occurs at the same time that valvular/vascular mineralization develops (Figure 2).

### 3.4. TNF Family of Cytokines

TNF-*α* strongly activates the canonical NF-*κ*B pathway and promotes the mineralization of VICs [33, 34]. TNF-*α* is first synthesized as a type II transmembrane protein, which upon cleavage by TNF-*α* converting enzyme (TACE) is released in the extracellular space [35]. It is produced by different cells including monocytes/macrophages, smooth muscle cells, and adipocytes among others. When stimulated, the ubiquitous TNF receptor 1 (TNFR1) activates and recruits TNFR-associated death domain (TRADD), which, in turn, recruits the Fas-associated protein with death domain (FADD) [36, 37]. As a result, FADD activates caspase-3 and caspase-8. Therefore, though not fully investigated, it is possible that TNF-*α*-induced mineralization of VIC culture is dependent on an apoptotic process. To this effect, apoptosis is one important process involved in the mineralization of the aortic valve [24]. Apoptotic bodies derived from plasma membrane and rich in ectonucleotidases promote the nucleation of hydroxyapatite and the formation of spheroid mineralized microparticles (2–5 *μ*m), which incidentally are the basic unit of mineralized material formed in CAVD [4, 38]. On the other hand, in response to TNF-*α*, TNFR1 may also recruit a receptor associated complex formed by TNF receptor associated factors (TRAF) and TRADD proteins, which activates transforming growth factor *β* activated kinase-1 (TAK1) [39]. TAK1 phosphorylates IKK*β* and triggers activation of NF-*κ*B. In vascular smooth muscle cells (VSMCs), TNF-*α* induces the expression of Msx2, a homeobox transcription factor involved in osteogenic transition [40]. Msx2 is increased in mineralized aortic valve and promotes activation of the Wnt pathway, which is by the way involved in the development of CAVD [41]. TNF-*α* has also crosstalk with interleukin-1*β* (IL-1*β*). In this regard, in mice deficient for the IL-1 receptor antagonist, IL-1RA, the circulating levels of TNF-*α* are increased and the aortic valve is thickened [42]. The double knockout mice IL-1RA^−/−^ TNF-*α* ^−/−^, however, do not develop fibrosis of the aortic valve. On the other hand, in isolated VICs, IL-1*β* induces the expression of MMP-1, suggesting that it may participate in the remodelling process of the aortic valve [43]. Hence, it is likely that IL-1*β* and TNF-*α* have reciprocal crosstalk, which promotes CAVD in mice. Recently, the expression of TNF-related apoptosis-inducing ligand (TRAIL) has been shown to be elevated in mineralized aortic valves [44]. TRAIL, a member of the TNF superfamily, has been shown to promote apoptosis-mediated mineralization of VICs through the death receptor 4, which is overexpressed by VICs during mineralization. Hence, overexpression of the TNF family of cytokines in CAVD plays an important role in promoting fibrosis/mineralization of the aortic valve through both apoptosis and an osteogenic program.

RANKL is expressed by activated CD4+, CD8+ cells, osteoblast, and bone marrow stromal cells. It is a type 2 transmembrane protein, which can be cleaved by metalloproteinase [45]. RANKL binds to its receptor RANK whereby TNFR-associated factors 2 and 6 (TRAFF2-6) are recruited [46]. This leads to a sequence of events that mediates the activation of AP-1, c-Src, and c-Cbl [47]. Osteoprotegerin (OPG) is a soluble decoy receptor for RANKL and is thus a negative regulator of RANKL signaling [48]. In bone, stimulation of RANK activates osteoclast activity. The OPG^−/−^ mice develop osteoporosis and vascular calcification [49]. In the aortic valve, a study based on immunodetection has revealed that RANKL is overexpressed in mineralized aortic valves, whereas the expression of OPG is decreased in the same valves [50]. In isolated VIC cultures, the stimulation of cells with RANKL activates mineralization and the expression of ALP [50]. In LDLR^−/−^ mice under high fat diet, the administration of OPG reduced mineralization of the aortic valve and decreased the expression of osteogenic genes, such as osterix and osteocalcin [51]. Taken together, these observations suggest that RANK signaling may play a permissive role in the development of CAVD.

### 3.5. Angiotensin II

The renin angiotensin system (RAS) is activated in patients with visceral obesity, a condition associated with the development of CAVD [32]. Studies have underscored that enzymes, which generate angiotensin II, are present in stenotic mineralized aortic valves [52]. Angiotensin converting enzyme (ACE) is present in human explanted stenotic aortic valves and colocalizes with low-density lipoprotein (LDL). Experiments with isolated LDL fraction showed that ACE was present in this fraction, suggesting that the angiotensin II-generating enzyme is possibly transported in the aortic valve. Moreover, mastocytes producing chymase, an angiotensin II-generating enzyme, are present in mineralized aortic valves and their density correlates with several indices of disease severity [53, 54]. As such, both ACE and chymase contribute to produce angiotensin II in the aortic valve. Moreover, Côté et al. identified in prehypertensive men with CAVD that the circulating levels of angiotensin II correlate with the valvular mRNA levels of TNF-*α* and IL-6 [55]. Moreover, immunohistological analyses revealed in stenotic aortic valves that angiotensin II was present in the vicinity of mineralized nodules and colocalized with TNF-*α* and IL-6 [55]. Patients under a therapy with angiotensin receptor blockers (ARBs) have a lower level of transcript encoding for IL-6 in their aortic valves [56]. Moreover, a retrospective study showed that ARBs are associated with a slower progression rate of aortic stenosis [57]. In mice, the administration of angiotensin II promoted the fibrotic remodelling of the aortic valve [58]. Also, the administration of olmesartan, an ARB, prevented the fibrotic remodelling of the aortic valve in the hypercholesterolemic rabbit [59]. Taken together, these data suggest that both systemic and valvular production of angiotensin II may contribute to CAVD. It is worth pointing out that angiotensin II is a potent activator of the NF-*κ*B pathway via the type 1 angiotensin receptor (AT1AR). The activation of NF-*κ*B by angiotensin II is relatively complex and differs from the signalling induced by TNF-*α*. Stimulation of AT1AR, a Gq protein coupled receptor, activates phospholipase C *β* (PLC*β*), which generates inositol triphosphate and diacylglycerol (DAG). In turn, DAG activates a typical protein kinase C (PKC). PKC phosphorylates an adaptor protein of the membrane guanylate kinase family, CARMA3 [60]. Next, activated CARMA3 forms a complex with Bcl10 and mucosa-associated lymphoid tissue lymphoma translocation 1 (MALT1), which deubiquitylates several members of the activating pathway of NF-*κ*B including IKK*γ*. As a result, the accumulation of IKK*γ* promotes the nuclear translocation of p65. Moreover, in VSMCs angiotensin II activates RhoA, which leads to the phosphorylation of p65 on Ser 536. It is suspected that NIK may mediate the phosphorylation of p65 on Ser 536 [61]. This sequence of events has possibly important repercussion as p65 Ser 536 is not inhibited by I*κ*B [62]. This creates a nuclear pool of p65 Ser 536, which is recycled with the chromatin-bound promoter [63]. Hence, this chain of events promotes a sustained activation of target genes of p65. However, whether a similar pathway of activation is present in VICs remains to be explored.

## 4. Innate Immune Response to Oxidized Lipid Species and Ectopic Mineralization of the Aortic Valve

### 4.1. Interaction between Lipid Retention and Toll-Like Receptors

The Toll-like receptors (TLRs), which are expressed by VICs, play a key role in driving the inflammatory reaction in response to several stimuli. In this regard, several lipid species can activate TLRs. Early investigations of CAVD have pointed out that apoB, oxidized-low-density lipoproteins (ox-LDL), and Lp(a) infiltrate mineralized aortic valves [64]. So far, several factors have been identified to play a role in lipid infiltration/retention of the aortic valve. To this effect, a high proportion of circulating small, dense, LDL has been associated with a higher accumulation of ox-LDL in the aortic valve and with an elevated level of TNF-*α* [10]. Small, dense LDLs have a higher oxidation rate and have a greater ability to infiltrate tissues. Of note, the small, dense LDL phenotype is one key feature of diabetes and the metabolic syndrome (MetS), two important risk factors for the development and progression of CAVD [65]. Also, during CAVD a higher production of proteoglycans (PGs) may help to promote the retention of lipids. The expression of decorin is increased in aortic valves and histological studies showed a colocalization of decorin with lipoprotein lipase (LPL), which is secreted by macrophages [66]. Studies have highlighted that interaction between LPL and decorin promotes the retention of LDL. The expression of biglycan is increased in human pathological samples of CAVD and participates in the retention of lipids within the aortic valve [67]. Moreover, emerging evidence indicates that biglycan is a potent agonist of TLRs. In this regard, in isolated VICs biglycan stimulates TLR-2, which leads to the secretion of phospholipid transfer protein (PLTP). In turn, PLTP may associate with apoA1 of high-density lipoproteins (HDLs), which may impede their function, namely, the reverse cholesterol transport (RCT) [67]. More recently, biglycan has been shown to promote the osteogenic transition of VICs through TLR-2, the extracellular signal-regulated protein kinase 1/2 (ERK1/2), and the NF-*κ*B pathways [68]. In isolated VICs, biglycan also induced the secretion of monocyte chemoattractant protein-1 (MCP-1) [69]. Also, it is worth underlining that ox-LDL has been shown to stimulate TLR-4, whereby the mineralization of VICs is promoted [70]. Zeng et al. reported in VICs that Notch1 promotes the activation of the NF-*κ*B pathway following stimulation of TLR-4 with bacterial lipopolysaccharide (LPS) [71]. The authors documented that the Notch intracellular domain (NICD) interacted with I*κ*B kinase*α* (IKK*α*) and in doing so promoted the nuclear translocation of p65 subunit of NF-*κ*B. However, the molecular mechanism whereby the NICD may impact on IKK and its phosphorylation status was not examined. The same group also identified that ox-LDL promoted the secretion of Jagged 1 by VICs, which ultimately led to increased levels of NICD and activation of an osteogenic response [72]. However, it is worth highlighting that frameshift mutations of the* NOTCH1* gene have been associated with bicuspid aortic valves (BAV) and mineralization of the aortic valve [73]. Investigations have shown that the NICD, which is the intracellular portion of the Notch1 receptor cleaved by the *γ*-secretase following stimulation of the Notch receptor, activates the expression of the hairy family of repressors that inhibit the expression of BMP2 and Runx-2, respectively, a bone morphogen and master transcription factor involved in osteogenesis [74]. Hence, Notch1 delivers signals that prevent the osteogenic transition of VICs. The apparent discrepancy between studies that have investigated the role of Notch in CAVD may result from different cell culture conditions and may be context dependent. Further work is needed to explore the interactions between Notch, inflammation, and the mineralization of the aortic valve.

The retention of lipids in the extracellular matrix of the aortic valve is also promoted by the elongation of GAG chains, which is enhanced by transforming growth factor *β*1 (TGF-*β*1) [75]. The retention of lipids in the aortic valve promotes, in turn, the oxidation of lipid species, which are potent agonists of the TLRs. During CAVD, the uncoupling of nitric oxide synthase (NOS) contributes to the increase of the oxidative stress [76]. Though the mechanisms of NOS uncoupling in the aortic valve have not been investigated, it is possible that a decreased bioavailability of NOS substrate, L-arginine, or cofactors, such as tetrahydrobiopterin, contribute to the increase of the production of ROS [77]. As a result, the increased production of ROS in mineralized aortic valves promotes the formation of lipid peroxidation products with proinflammatory activities. Recently, pathways leading to the production of highly reactive lipid species, derived from ox-LDL, have been identified and may play an important role in the immune response and the mineralization of the aortic valve.

### 4.2. Lp(a), Lipoprotein-Associated Phospholipase A2, and Inflammation-Mediated Mineralization of the Aortic Valve

In a genome-wide association study (GWAS), Thanassoulis et al. have recently identified that single nucleotide polymorphisms (SNPs) of the* LPA *gene encoding for Lp(a) were associated with CAVD [78]. Using a Mendelian randomization study design two other independent studies have since then corroborated the positive association between Lp(a) and CAVD [79, 80]. These studies thus suggest a causal role for Lp(a) in the development of CAVD. Though the physiological role of Lp(a) is largely unknown, it is well accepted that it is a major carrier of oxidized-phospholipids (ox-PLs) in the blood plasma [81]. Ox-PLs are potent stimulators of inflammation through their metabolism by phospholipase enzymes [82]. Mahmut et al. have recently discovered that lipoprotein-associated phospholipase A2 (Lp-PLA2) is overexpressed in mineralized aortic valve [83]. Immunohistological analyses suggest that Lp-PLA2 is transported in the aortic valve by lipoproteins and also secreted by infiltrating macrophages. The transcript level of Lp-PLA2 in stenotic aortic valves correlated with several indices of tissue remodelling and mineralization of the aortic valve. Of note, ox-PLs are the natural substrates for Lp-PLA2 and are hydrolyzed into lysophosphatidylcholine (LPC), which are highly reactive and proinflammatory. In isolated VICs, LPC promoted the expression of ALP, ENPP1, and the sodium-phosphate cotransporter Pit1 [83]. Of note, the expressions of ALP, ENPP1, and Pit1 are increased in mineralized aortic valves and contribute actively to the mineralization process [28]. Hence, it is likely that ox-PLs transported by Lp(a) are transformed into LPC by Lp-PLA2 in the aortic valve, whereby inflammation and mineralization are triggered.

### 4.3. Lipoxygenase and Leukotrienes: Role in CAVD

Arachidonate is a polyunsaturated fatty acid (20 : 4), which is produced from phospholipids and the action of PLA2 and lipoprotein lipase (LPL), which are incidentally overexpressed in the aortic valve during CAVD [66, 84]. Arachidonate is a powerful metabolite that exerts an important control over inflammation. In mineralized aortic valve the expressions of 5-lipoxygenase (5-LO) and LTC4S are increased and correlate with echocardiographic indices of aortic stenosis severity [85]. Moreover, the expression of 5-LO was documented in both macrophages and VICs. The combined action of 5-LO and LTC4S generates leukotriene C(4) (LTC4), which has potent proinflammatory activities [86].* In vitro*, LTC4 induced oxidative stress in VICs with the loss of mitochondrial membrane potential and the concomitant expression of BMP2 and BMP6 [85]. Hence, the expression of 5-LO by VICs promotes the production of leukotrienes, which constitute short acting messengers that drives inflammation of tissues. Whether the 5-LO pathway plays a crucial role in the mineralization of the aortic valve remains to be investigated.

### 4.4. Remodelling, Neoangiogenesis, and MMPs

The activation of innate immunity leads to the expression of several factors involved in tissue remodelling. TGF-*β*1, which is overexpressed in CAVD, activates VICs and promotes their transformation into secretory myofibroblast-like cells [87]. Of interest, the inhibition of serotoninergic receptor 5-HT_2B_ prevents TGF-*β*1-induced transformation of VICs into myofibroblast [88]. This latter finding may have pathobiological significance, since mast cells are present in mineralized aortic valves and may thus affect TGF-*β* signalling through the production of serotonin. One simple observation of explanted human aortic valves is that neoangiogenesis is associated with the development of CAVD. Of note, the density of neovessels correlates with the presence of chronic inflammatory infiltrates [6]. The expression of heat shock protein 60 (HSP60), a marker of inflammation, correlates with the development of neoangiogenesis in stenotic aortic valves [89]. In mineralized aortic valves, endothelial progenitor cells CD34+ are present in valvular tissue [90]. In mice defective for chondromodulin-1, a protein with antiangiogenic properties, there is a neovascularization of the aortic valve and mineralization of leaflets [91]. Though the role of angiogenesis in CAVD is not elucidated yet, one hypothesis is that it contributes to the recruitment of inflammatory cells in a positive feedback loop. Osteonectin (SPARC), a matricellular protein highly expressed in stenotic aortic valves, is cleaved by metalloproteinases into a proangiogenic peptide [90]. The expression of several MMPs, including MMP2-3 and MMP9, is elevated in mineralized aortic valves [92]. In addition, a recent study has identified that MMP12, which is a potent elastase, is overexpressed in stenotic aortic valves [93]. Also, in human mineralized aortic valves the expression of cathepsins K, V, and S is increased [94]. In apoE^−/−^ mice with 5/6 nephrectomy, cathepsin S induced elastolysis and promoted the mineralization of the aortic valve [95]. These studies thus suggest that during the remodelling process elastic fibers fragments with osteogenic properties are generated and may participate in the mineralization of the aortic valve.

## 5. Adaptive Immunity

From the above discussion it is clear that there is an activation of innate immunity in CAVD. Though it has been observed more than a decade ago that CD4+ and CD8+ T cells infiltrate the aortic valve during CAVD, it is only recently that investigations have shown a clonal expansion of T cells in mineralized aortic valves. In a seminal work, Winchester et al. showed that the proportion of circulating CD3+ T cells expressing HLA-DR was increased in subjects with CAVD [96]. Also, the proportion of circulating CD8+ CD57+ T cell subset expressing HLA-DR was elevated during CAVD. These findings thus suggest that a subset of memory T cells is activated in patients with CAVD. In mineralized aortic valves, both bicuspid and tricuspid, the clonal expansions of the TCR repertoire were documented. Immunohistological analyses of explanted mineralized aortic valves showed that CD8+ CD28 null cells were present at the proximity of mineralized nodules [96, 97]. These data thus suggest that a systemic adaptive immunity, coupled to lymphocytic infiltration of the aortic valve, is activated during CAVD. Though the antigen(s) responsible for this response in CAVD remains to be determined, it is possible that oxidatively modified epitopes may play a role [98]. Several questions though remain to be studied with regard to the role of adaptive immunity in the development of CAVD. The identification of epitopes that induce an adaptive immune response, as well as the role of this response in fibrocalcific remodelling of the aortic valve, remains to be explored.

## 6. An Integrative View of Inflammation in CAVD and Potential Therapeutic Opportunities

Several factors may promote inflammation in CAVD, but an important culprit is possibly the oxidized lipids [99]. In this regard, ox-LDL is a potent trigger of inflammation through the Toll-like receptors. Also, ox-LDL generates epitopes, which may activate adaptive immunity [100]. In addition, the overexpression of Lp-PLA2, LPL, and 5-LO in CAVD contributes to generate bioactive lipid-derived species, which amplify inflammation. These factors along with angiotensin II contribute to the activation of the NF-*κ*B cascade. The activation of NF-*κ*B in CAVD is substantiated by an elevated level of phosphorylated I*κ*B*α* (Ser32) along with the overexpression of target genes such as IL-6 [23]. In this scheme of things, it is possible that NF-*κ*B represents a hub of signalling, which may drive, at least in part, the fibrocalcific remodelling process of the aortic valve. Different therapeutic alternatives could be examined in order to prevent the progression of CAVD. In this regard, investigations to decrease activation of the RAS or blocking enzyme pathways that promote the production of highly reactive lipid species are needed. The recent discovery that IL-6 may represent an effector cytokine in promoting the osteogenic transition of VICs warrants further investigation. The use of monoclonal antibodies directed against TNF-*α* or IL-6 could be examined in preclinical animal models. The cardiovascular inflammation reduction trial (CIRT) will examine if the administration of methotrexate can reduce cardiovascular events [101]. This study will randomize 7000 patients to a placebo or methotrexate. Though this study is not designed and powered to study CAVD, it will examine as a secondary outcome the rate of CAVD in both arms. Though the mechanism by which methotrexate reduces inflammation remains, to some extent, obscure, in the hypercholesterolemic rabbit, it decreased the size of atherosclerotic plaques and* in vitro* it reduced the expression of TNF-*α*, IL1*β*, and CXCL2 in human umbilical vein endothelial cells treated with TNF-*α* [102]. The repositioning of drugs with an anti-inflammatory effect in CAVD is a potential therapeutic avenue, which needs further exploration [103]. However, basic and translational works are clearly needed in this field in order to tease out the key underpinning processes that link immunity with CAVD.

## 7. Conclusion

Research in the last several years has clearly identified that CAVD is an active disorder, which has an immune component. Both innate and adaptive immunity are activated during CAVD. Several upstream factors converge on the NF-*κ*B. Oxidized lipid species and angiotensin II promote activation of the NF-*κ*B cascade, which increases the expression of different cytokines. Both apoptosis-mediated mineralization and osteogenic transition of VICs are activated by NF-*κ*B and promote the ectopic mineralization of the aortic valves. Recent discoveries linking the immune response with CAVD should spur more translational work in order to develop novel therapeutic alternatives for this chronic process affecting our aging societies.
